# Navigating oxygen deprivation: liver transcriptomic responses of the red eared slider turtle to environmental anoxia

**DOI:** 10.7717/peerj.8144

**Published:** 2019-11-26

**Authors:** Kyle K. Biggar, Jing Zhang, Kenneth B. Storey

**Affiliations:** 1 Institute of Biochemistry & Department of Biology, Carleton University, Ottawa, Ontario, Canada; 2 The hospital for sick children, Neuroscience and Mental Health, Toronto, Ontario, Canada

**Keywords:** Anoxia tolerance, Hypometabolism, Metabolic rate depression, Red eared slider turtle, DNA damage response

## Abstract

The best facultative anaerobes among vertebrates are members of the genera *Trachemys* (pond slider turtles) and *Chrysemys* (painted turtles), and are able to survive without oxygen for up to 12 to 18 weeks at ∼3 °C. In this study, we utilized RNAseq to profile the transcriptomic changes that take place in response to 20 hrs of anoxia at 5 °C in the liver of the red eared slide turtle (*Trachemys scripta elegans*). Sequencing reads were obtained from at least 18,169 different genes and represented a minimum 49x coverage of the *C. picta bellii* exome. A total of 3,105 genes showed statistically significant changes in gene expression between the two animal groups, of which 971 also exhibited a fold change equal to or greater than 50% of control normoxic values. This study also highlights a number of anoxia-responsive molecular pathways that are may be important to navigating anoxia survival. These pathways were enriched in mRNA found to significantly increase in response to anoxia and included molecular processes such as DNA damage repair and metabolic reprogramming. For example, our results indicate that the anoxic turtle may utilize succinate metabolism to yield a molecule of GTP in addition to the two molecules that results from lactate production, and agrees with other established models of anoxia tolerance. Collectively, our analysis provides a snapshot of the molecular landscape of the anoxic turtle and may provide hints into the how this animal is capable of surviving this extreme environmental stress.

## Introduction

Maintaining optimal and continuous oxygen supply to organs (in order to maintain high rates of aerobic metabolism) is not universal across the animal kingdom. Many facultative anaerobic organisms, including a variety of ectothermic vertebrates, are able to survive extended periods without a supply of oxygen (Biggar, Groom & Storey, 2012; Storey, 2007; Nilsson & Renshaw, 2004). For example, a lack of available oxygen is common among turtles, which spend the majority of their lives diving underwater (Clark & Miller, 1973; Jackson, 1968). For bouts of short-term oxygen deprivation, such as during underwater dives for food, simply increasing anaerobic metabolism is able to meet metabolic demands (Storey & Storey, 2004). However, for long term survival, such as overwintering in ice-locked ponds and waterways, turtles must resort to at least one of two established modes of survival: extrapulmonary modes of oxygen uptake, or a severe reduction in metabolic demand such that anaerobic metabolism can meet energetic demand (i.e., hypometabolism).

The best facultative anaerobes among vertebrates are members of the genera *Trachemys* (pond slider turtles) and *Chrysemys* (painted turtles). These turtles are able to survive without oxygen for up to two weeks at ∼16 °C and for 12 to 18 weeks at ∼3 °C (Ultsch, 1985; Ultsch & Jackson, 1982). By comparison, mammalian tissues are notoriously sensitive to even brief episodes of anoxia; for example, humans can endure only a few minutes of anoxia without irreversible brain damage. Previously reported by Hochachka et al. (1996), the main features of anoxia survival via hypometabolism include: (1) a mechanism of oxygen sensing such that anoxic transitions can be signaled to cells, (2) a set of pro-survival genes that are up-regulated to help coordinate adaptation to the hypometabolic state, (3) a set of non-essential genes and (4) non-essential pathways that are down-regulated to conserve energy stores, (5) reduction in activity and impulse frequency in neural tissues, and (6) a sustainable re-balance of metabolism such that cellular energy demands can be met by anaerobic energy production (Hochachka, Land & Buck, 1997). Collectively, these are thought to be fundamental, and highly regulated, processes that allow turtles to survive extended periods of reduced oxygen in a hypometabolic state. In turtles, a profound metabolic rate depression to only 10–20% of the corresponding aerobic resting rate, at the same temperature, occurs in response to anoxia (Storey & Storey, 1990). Indeed, to help establish this metabolically depressed state, it has been well established that turtles respond to anoxia with a suit of cellular modifications that involve transcriptional, translational, post-translational (i.e., reversible protein phosphorylation), as well as epigenetic, and microRNA-dependent post-transcriptional regulations (Storey, 2007; Krivoruchko & Storey, 2015; Biggar & Storey, 2017).

Upon encountering hypoxic conditions, the first response of the turtle is to enhance oxygen delivery and the oxygen extraction systems. If oxygen concentrations continue to fall, the systemic alterations to oxygen extraction quickly become inadequate to supply enough oxygen to deprived tissues. When this occurs, oxygen-independent metabolic pathways, such as anaerobic glycolysis, are fully recruited and are followed by regulatory actions that reduce oxygen demand by cells (Hochachka et al., 1996). This introduces a very important issue—increasing the rate of glycolysis does increase ATP output, but also results in a quick depletion of internal fuel reserves, as well as a large accumulation of acidic end products that must be dealt with (Kelly & Storey, 1988). One predominant component of ATP conservation to match the supply provided by anaerobic glycosis is the suppression of protein synthesis.

Protein synthesis consumes a very large portion of available ATP turnover in cells under normoxic conditions, requiring about 5 ATP equivalents per peptide bond of a newly synthesized protein (Cramer et al., 1991). Some freshwater turtles have been shown to decrease the rate of ATP use by suppressing protein synthesis to as low as 6% during anoxia (Hochachka et al., 1996). Several studies have explored the *in vivo* protein synthesis rates during anoxia-induced metabolic depression in turtles (Brooks & Storey, 1993; Land & Hochachka, 1994; Fraser et al., 2001). Such a dramatic regulation of the transcriptome during hypometabolism might be achieved in several ways: (1) a reduction of the amount of mRNA transcripts available (i.e., changes in mRNA expression), (2) differential regulation of the assembly and activity of ribosomal translational machinery, (3) epigenetic-level gene regulation, and (4) microRNA interference.

To date, a large body of literature has established that in response to anoxia, the red-eared slider turtle is able to respond to oxygen lack through a number of molecular mechanisms that include; (1) the expression of various molecular chaperones, (2) increased antioxidant defense, (3) stress-responsive transcription factors, (4) pro-survival proteins, and (5) a significant restructuring of metabolism such that ATP demand can be met in the new anaerobic environment. For example, one recent study documented epigenetic changes that dynamically occur on histone proteins to alter the chromatin state in a manner that is responsive to anoxia. This suggests chromatin-level regulation of gene expression in an anoxia-responsive manner in the turtle (Wijenayake, Hawkins & Storey, 2018). Given the large amount of supporting knowledge behind the molecular mechanisms that permits this turtle to survive extended periods of anoxia, we chose the red-eared slider turtle as the most appropriate model for this study. Supplemented with the recent sequencing of the western painted turtle genome (*C. picta bellii*), an unprecedented degree of insight can now be gained into the transcriptomic response behind turtle anoxia tolerance in general (Shaffer et al., 2013; Keenan et al., 2015; Hong et al., 2019). In the present study, we utilize this genomic resource for several purposes that include: (1) to investigate whether anoxic-responsive changes in the transcriptome are reflective of known physiological responses, (2) to provide insight into transcriptome-wide changes in biological processes and molecular pathways that are sensitive to anoxia, and (3) to identify new targets for future functional study.

## Material and Methods

### Animal care and treatment

Adult female red-eared sliders (*T. s. elegans*), 700–1,500 g, were acquired from local suppliers (Ottawa, Ontario, Canada) and held at 5 ± 1 °C in large 50 L plastic tanks (2 turtles per tank) filled with dechlorinated tap water for an average of 7–8 days before use. Control (normoxic) turtles were sampled from this condition. For anoxia exposure, turtles were transferred to large buckets at 5 ± 1 °C that had been previously bubbled with N_2_ gas for 1 hr; 2 turtles were added per bucket in 30 min intervals to allow for sufficient sampling and post-processing time between individuals. Bubbling was continued for 1 hr after the last turtle was added and was reinitiated again during sampling of the animals. A wire mesh was fitted into the tank about 5 cm below the water surface so that turtles remained submerged throughout the 20 hrs experimental anoxia exposure. All animals were killed by decapitation and liver tissue was rapidly dissected out, frozen in liquid nitrogen and stored at −80 °C until use. The time-course of anoxia exposure was chosen as described in Jackson (2002). All animals were cared for in accordance with the guidelines of the Canadian Council on Animal Care and all experimental procedures had the prior approval of the Carleton University Animal Care Committee (IACUC approval #B09-20).

### RNA isolation and library preparation

Total RNA was isolated from the liver tissue of control and 20 hrs anoxic turtles (*n* = 3 individual turtles) using Trizol (Invitrogen; Cat#15596-018). Briefly, 100 mg of tissue was homogenized in 1 mL Trizol using a Polytron homogenizer followed by the addition of 200 µL of chloroform and centrifugation at 10,000×g for 15 min at 4 °C. The upper aqueous layer (containing RNA) was removed and collected into an RNAse-free microcentrifuge tube. Total RNA was then precipitated with the addition of 500 µL of isopropanol followed by incubation for 10 min at room temperature. Samples were then centrifuged again as above. The RNA pellet was washed with 70% ethanol. Samples were loaded onto Qiagen RNeasy filter columns, and kit protocol was followed according to manufacturer’s instructions, including an on-column DNase step to remove any residual contaminating DNA. RNA integrity and concentration were assessed using the Agilent 2100 Bioanalyzer (Santa Clara, CA, USA) at the McGill University and Genome Quebec Innovation Centre (Montreal, QC). RNA Integrity Number (RIN) values for total RNA used for sequencing were greater than 7. Libraries were constructed with the TruSeq Stranded mRNA Sample Preparation Kit (Illumina) following manufacturers guidelines.

### Transcriptome sequencing and assembly

The RNA sequencing performed at the McGill University and Genome Quebec Innovation Centre (Montreal, QC) generated 148 to 178 million paired reads per library using a Illumina HiSeq 2500 T1 sequencer. Trimming and clipping of Illumina sequencing adapters and other adapters was performed by the sequencing facility using Trimmomatic software (Bolger, Lohse & Usadel, 2014). Reads are trimmed from the 3′end to have a phred score of at least 30. Illumina sequencing adapters are removed from the reads, and all reads are required to have a length of at least 32. The filtered reads were aligned to the Chrysemys_picta_bellii-3.0.3 reference genome (NCBI) using TopHat software (Trapnell, Pachter & Salzberg, 2009). TopHat splits reads to align them across known and novel splice junctions. The filtered and aligned *T. scripta elegans* RNAseq dataset represents a minimum 49x coverage of the *C. picta bellii* exome. To estimate transcript and gene abundances, Cufflinks software (v1.3.0) was used (Trapnell et al., 2013), generating normalized FPKMs (Fragments Per Kilobase of exon model per Million mapped fragments) for each annotated gene. All trimming and alignment statistics are provided in Table 1. All raw sequencing reads are able available through the NCBI sequence read archive database (SRA PRJNA525986). The GFF used for alignment is available at https://www.ncbi.nlm.nih.gov/bioproject/PRJNA78657/.

**Table 1:** Trimming and alignment statistics. Alternative alignments indicates the number of duplicate read entries providing alternative coordinates.

**Sample**	**Raw reads**	**Surviving reads**	**%**	**Aligned reads**	**%**	**Alternative alignments**	**%**	**Coverage**	**Exonic rate**	**Genes**
C-1	157,384,476	153,070,220	97	96,895,249	63	11,227,181	12	63	0.73	19,230
C-2	177,029,988	171,370,576	97	101,933,083	59	10,532,358	10	63	0.75	18,500
C-3	160,902,862	155,600,064	97	91,604,554	59	9,145,947	10	57	0.75	18,309
20-1	161,001,556	155,718,808	97	85,453,907	55	8,975,060	11	54	0.74	18,178
20-2	148,150,044	143,300,486	97	78,889,776	55	8,167,035	10	51	0.74	18,169
20-3	150,765,402	145,901,344	97	80,739,759	55	8,289,079	10	49	0.74	18,252

### Gene differential expression (DE) analysis

Normalized expression values were subject to unsupervised hierarchical cluster analysis. A heat map was used to visualize the clustering results and the overall gene expression pattern for each experimental condition and individual animal sample. All raw FPKM values were transformed through log_10_(FPKM+1) prior to plotting. The R package *gplots* (Warnes et al., 2016) with a custom workflow was used for generating the heat map. Volcano distribution was used to visualize the DE (i.e., individual gene level) results. The log_2_ transformed fold changes were plotted against –log10 transformed *p*-values (false discovery rate, or FDR, adjusted) for each gene.

### Gene set (GS) analysis

Gene Ontology (GO) term and KEGG pathway (Ashburner et al., 2000; Kanehisa et al., 2016) were used for GS analysis. In order to explore the molecular events responsible for the potential anoxia-responsive adjustments to the cell, both biological processes (BP) and molecular function (MF) sets were used for GO term analysis. KEGG gene sets were used to further explore the pathways affected by the anoxic conditions and generate visual representations with the integration of the relevant DE results.

Gene level statistics of all genes were used for GS analysis, as opposed to only significant genes (i.e., the contingency table method). As such, the current method was able to take the overall gene expression profile into consideration for GS enrichment without relying on the univariate analysis significance threshold. By combining gene level *p*-values with directional statistics (e.g., fold change or t-statistics), the present GS assessment provided both directional and non-directional GS statistics. Specifically, non-directional, distinct directional and mixed directional tests were conducted on both GO term and KEGG GS analyses. With the distinct-directional analysis, we were able to explore the overall anoxia response of the pathways, as the test weighs the opposite directional changes exhibited by subsets of the genes, thereby determining the direction in which the gene sets are regulated. The mixed directional test investigates the directional impact to the pathways from the subsets of the genes included in the GS based on DE statistics. For example, a pathway can be regulated in both positive and negative directions if the two opposite directional regulations on two subsets of genes significantly affect the GS statistics. For both the distinct and mixed directional tests, GS level *p*-values were calculated based on the directionality exhibited in the gene level statistics. The non-directional GS analysis identified gene sets that were significantly impacted by the anoxia stress solely based on the significance test results (*p*-values) from the DE results.

To minimize analysis bias, several GS enrichment methods were used to comprehensively and thoroughly assess the biological significance of anoxia-responsive gene expression profiles through a consensus score-based ranking method (Väremo, Nielsen & Nookaew, 2013). A total of eight enrichment methods were used, including parametric (Fisher’s combined probability, Stouffer’s test, Reporter features test, and parametric analysis of gene set enrichment (i.e., PAGE) test) (Patil & Nielsen, 2005; Kim & Volsky, 2005), non-parametric (Tail strength test, Wilcoxon rank-sum test, and gene set enrichment analysis (i.e., GSEA) test) approaches (Taylor & Tibshirani, 2006; Smyth, 2005; Subramanian et al., 2005), as well as a maxmean method developed by Efron and Tibshirani (Efron & Tibshirani, 2007). Gene sampling permutation test (x1000) was used for generating the null distribution for all enrichment methods to determine the significance of the enrichment results (Goeman & Buhlmann, 2007). All the GS *p*-values from the individual enrichment methods were adjusted by FDR. Consistent with the DE analysis, best *p*-value was set for the gene level statistics for repeated genes. All the gene sets were ranked for individual enrichment tests using the associated *p*-values. The median value of the ranks was considered the consensus score (normalized to a 0–100 scale) for each GS.

**Figure 1:**
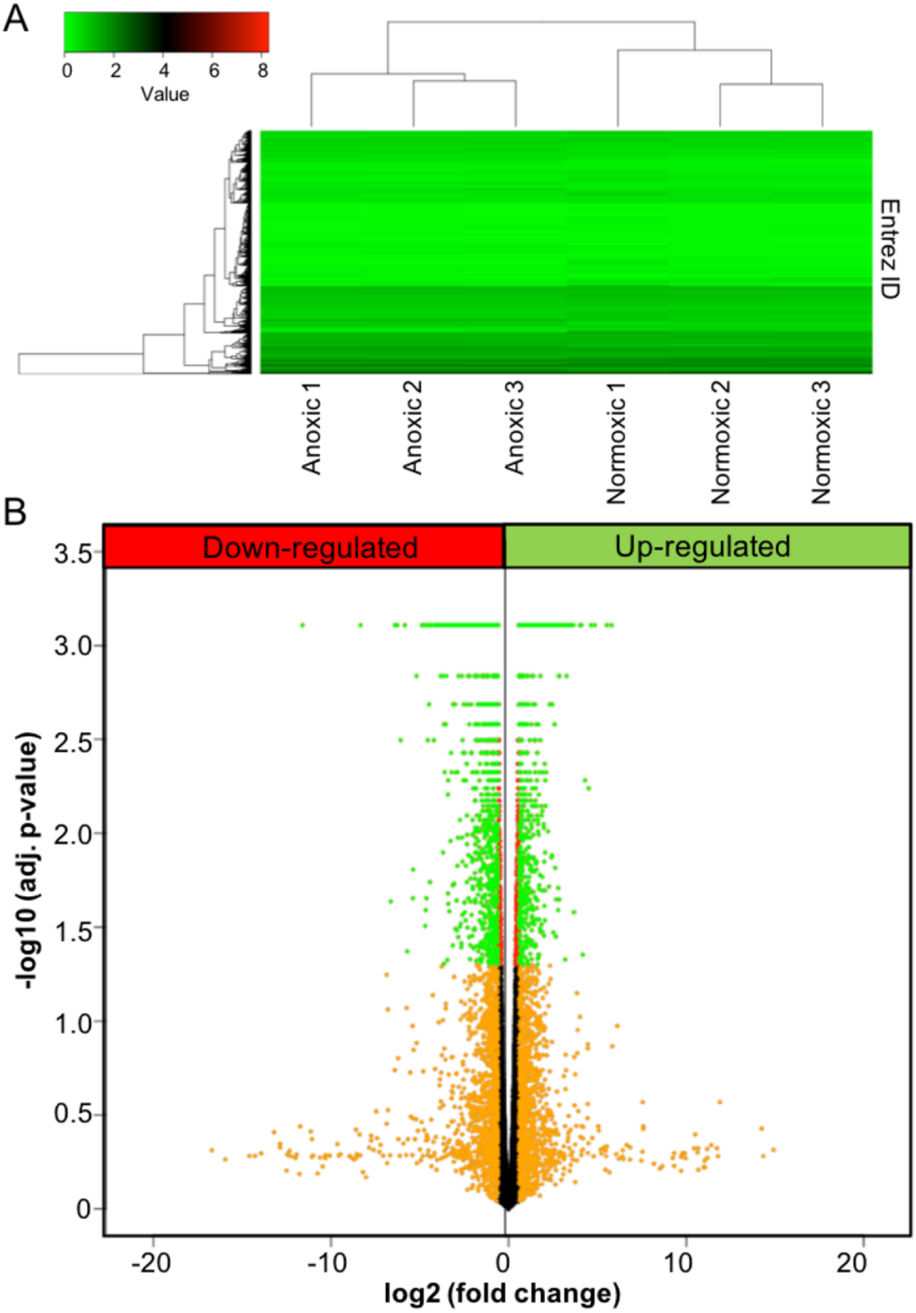
Anoxia-induced transcriptomic changes. (A) Heat map showing both the results for unsupervised hierarchical clustering analysis and overall gene expression pattern. FPKM values were plotted after log_10_(FPKM+1) transformation. (B) A volcano plot showing the results for DE analysis. Logarithmic transformed p-values (FDR corrected) and fold changes are plotted. Orange colour represents the fold changes equal or greater than 1.5; red represents *p*-values less than 0.05; green represents the genes that meet both criteria.

Quadrant scatterplot and boxplot were used to visualize the results for GS analysis for all five directionality classes. Specifically, the median GS *p*-value (cut-off: 0.1) were calculated based on the multiple GS enrichment tests and plotted against the GS consensus score in the boxplots, and used as the consensus and final *p*-value for the gene sets tested. Gene sets with a final *p*-value less than 0.05 were considered significantly altered by anoxia. Additionally, gene sets from either GO term or KEGG pathways were sorted according to the consensus score (cut-off: 50) and plotted using boxplot. Furthermore, selected pathways were visualized with the related DE results. The R package *pathview* (Luo & Brouwer, 2013) was used for ID conversion and KEGG pathway visualization. GS enrichment tests and consensus score processing was conducted using the R package *piano* (Väremo, Nielsen & Nookaew, 2013) with a custom workflow specific to the current study. Results visualization for GS analysis was carried out using R packages *reshape2* (Wickham, 2007) and *ggplot2* (Wickham, 2009) with customization.

## Results

### Gene differential expression (DE) analysis

To investigate the effects of anoxia on the transcriptomic responses of the liver, adult turtles were submerged in anoxic water for 20 hrs at 5 °C, and the liver tissue was sampled and analyzed using RNA-seq. A heat map was used to visualize unsupervised hierarchical clustering and overall gene expression profiles (Fig. 1A). While variance existed between individual samples in the same experimental group, the clustering analysis demonstrated a clear separation of the overall gene expression pattern between control and anoxic turtles. A volcano plot was used to visualize DE analysis results (Fig. 1B). A total of 3,105 genes showed statistically significant changes in gene expression between the two animal groups, of which 971 also exhibited a fold change equal to or greater than 50% of control normoxic values. The plot uses red colour for genes with an adjusted *p*-value less than 0.05 (statistically significant), orange for fold changes greater than 50% of control normoxic values, and green for the genes that meet both thresholds. Within this dataset, expression of 64 genes increased greater than 5-fold when compared to control values (Table 2), while 417 genes decreased to less than 20% of control values. All fold changes in gene expression are listed as Table S1.

**Table 2:** FPKM values measured for genes found to increase greater than 5-fold in response to anoxia.

**Fold change**	**Gene name**	**Gene ID**	**Locus**	**Mean FPKM (Control)**	**Mean FPKM (Anoxia)**
5.01	Heterogeneous nuclear ribonucleoprotein C	gene16690	NW_007281930.1:7005-18045	1.00	4.64
5.12	Cholesteryl ester transfer protein	gene3179	NC_024229.1:1268894-1284850	1.51	7.48
5.25	Condensin-2 complex subunit H2	gene12017	NW_007281530.1:1767412-1786677	1.53	7.88
5.25	V-type proton ATPase subunit S1-like protein	gene3838	NW_007281339.1:7340969-7369713	0.21	1.03
5.28	Centrosomal protein of 131 kDa	gene4049	NW_007281340.1:11239101-11269228	0.43	2.07
5.28	Phosphoserine phosphatase	gene20113	NW_007359849.1:8379950-8401297	0.86	4.40
5.30	Tubulin polyglutamylase TTLL6	gene21468	NW_007359872.1:2397248-2424904	0.05	0.25
5.30	RNA helicase Mov10l1	gene1061	NC_024219.1:57385859-57457741	0.11	0.64
5.34	DNA (cytosine-5)-methyltransferase 3-like	gene7912	NW_007281399.1:3261284-3279562	0.06	0.34
5.41	Mitochondrial fission regulator 2	gene1488	NC_024220.1:49284333-49299028	0.87	5.47
5.42	Caspase recruitment domain-containing protein 9	gene6122	NW_007281364.1:6381680-6402673	0.23	1.12
5.52	Ectodysplasin-A receptor-associated adapter protein	gene6427	NW_007281370.1:1143933-1158884	0.36	1.80
5.53	Apoptosis-inducing, TAF9-like domain 1	gene3583	NC_024234.1:6047045-6056630	0.77	4.12
5.63	NADPH oxidase 1	gene2721	NC_024225.1:13666090-13681627	0.07	0.39
5.70	Occludin	gene3858	NW_007281339.1:8859408-8890164	0.85	4.48
5.73	Huntingtin-interacting protein 1	gene12854	NW_007281570.1:421065-557723	0.55	3.36
5.75	Mitochondrial folate transporter/carrier	gene10067	NW_007281454.1:2041943-2060033	0.56	3.19
5.83	Exportin-T	gene8389	NW_007281409.1:517438-594831	1.21	6.65
5.91	Probable C-mannosyltransferase DPY19L1	gene10618	NW_007281477.1:1919781-2027394	0.64	3.56
5.98	Ras-related protein Rab-26	gene23555	NW_007359899.1:14273841-14485260	0.82	4.25
6.02	Transmembrane protein 218	gene8138	NW_007281402.1:4074561-4089102	0.38	2.05
6.06	Cilia- and flagella-associated protein 251	gene22360	NW_007359884.1:8972215-9006606	0.06	0.39
6.15	Transient receptor potential cation channel subfamily V member 1	gene5628	NW_007281358.1:4608333-4641788	0.14	0.87
6.22	T-box transcription factor TBX6	gene15903	NW_007281789.1:246506-252000	0.33	1.98
6.31	Prominin-1	gene11980	NW_007281529.1:386175-473658	0.10	0.64
6.39	Breast carcinoma-amplified sequence 1	gene9931	NW_007281448.1:1292657-1386845	0.10	0.69
6.40	HERV-H LTR-associating protein 1	gene23918	NW_007359902.1:15300963-15351050	0.14	0.91
6.42	TBC1 domain family member 16	gene4064	NW_007281340.1:12087218-12134952	1.63	9.92
6.47	Sulfotransferase	gene22400	NW_007359884.1:10968341-11116484	0.25	1.54
6.81	Putative sodium-coupled neutral amino acid transporter 8	gene3200	NC_024229.1:2867872-2904393	0.07	0.50
6.91	Polypeptide N-acetylgalactosaminyltransferase	gene19712	NW_007359841.1:19853527-20196374	0.07	0.48
6.96	Mitochondrial enolase superfamily member 1	gene19866	NW_007359845.1:5442404-5483366	1.96	13.11
7.08	Apical endosomal glycoprotein	gene11563	NW_007281514.1:751300-805295	0.04	0.27
7.15	Homogentisate 1,2-dioxygenase	gene108	NC_024218.1:16807818-16855000	0.26	1.82
7.24	Ankyrin repeat domain-containing protein 50	gene6263	NW_007281367.1:20195-70713	0.42	2.96
7.29	Hemicentin-2; Receptor protein-tyrosine kinase	gene5857	NW_007281360.1:4685366-4806403	0.53	3.90
7.30	Oncostatin-M-specific receptor subunit beta	gene5251	NW_007281354.1:2634377-2714975	0.86	6.38
7.39	Intermediate conductance calcium-activated potassium channel protein 4	gene15763	NW_007281776.1:338903-352616	0.16	1.22
7.43	E3 ubiquitin-protein ligase MSL2	gene11846	NW_007281524.1:1835898-1890751	0.29	1.86
7.76	NHS-like protein 2	gene13609	NW_007281610.1:13311-187817	0.47	3.20
8.35	Tetratricopeptide repeat protein 31	gene12423	NW_007281546.1:900243-921746	0.97	7.87
8.35	Transmembrane protein 240	gene3643	NC_024234.1:10334661-10371762	0.12	0.98
9.11	GTP-binding protein Di-Ras3	gene2948	NC_024225.1:40419019-40426191	0.04	0.32
9.21	Ectonucleotide pyrophosphatase/phosphodiesterase family member 1	gene1524	NC_024220.1:52582788-52659285	0.05	0.40
9.45	Integrin alpha-E	gene5634	NW_007281358.1:4706392-4771983	0.45	4.04
9.60	Regulator of G-protein signaling 9-binding protein	gene3306	NC_024229.1:16667911-16670807	0.03	0.29
9.62	Dpy-19 Like C-Mannosyltransferase 1	gene10618	NW_007281477.1:1919781-2027394	1.46	14.31
9.93	Potassium voltage-gated channel subfamily V member 2	gene2222	NC_024223.1:12230785-12238276	0.05	0.47
10.41	Serine/threonine-protein kinase NIM1	gene5286	NW_007281354.1:7715828-7732336	0.07	0.76
11.11	Fibronectin type III domain-containing protein 4	gene15588	NW_007281760.1:51390-91029	2.19	23.79
11.68	Estradiol 17-beta-dehydrogenase 2	gene5112	NW_007281352.1:6859463-6870938	0.07	0.80
11.75	Pannexin	gene8126	NW_007281402.1:2761308-2777293	0.05	0.61
12.25	Nebulin	gene8448	NW_007281410.1:3153891-3365730	0.09	1.07
12.42	Perilipin	gene5450	NW_007281356.1:5936040-5951851	0.32	3.73
12.82	Leucine-rich repeat-containing protein 38	gene3545	NC_024234.1:4003997-4030353	0.03	0.34
16.22	Tyrosine-protein kinase; Tyrosine-protein kinase ABL2	gene2786	NC_024225.1:22587197-22668610	0.54	8.08
17.04	Glucokinase regulatory protein	gene17516	NW_007282306.1:7094-42264	0.64	10.68
18.09	Tripartite motif-containing protein 16; E3 ubiquitin-protein ligase TRIM16	gene22614	NW_007359887.1:6894167-6907028	0.02	0.38
19.71	EF-hand calcium-binding domain-containing protein 3	gene13683	NW_007281615.1:460307-476141	0.04	0.69
22.70	Guanylyl cyclase-activating protein 2	gene8849	NW_007281419.1:2399418-2408781	0.14	3.06
24.76	Contactin-associated protein-like 2	gene20384	NW_007359856.1:12726798-14405054	0.05	1.07
28.83	F-box only protein 2	gene3570	NC_024234.1:4909708-4916629	0.05	1.46
46.44	WD repeat-containing protein 27	gene1362	NC_024220.1:30833693-31040151	0.04	2.05
55.88	Transmembrane protein 198	gene14453	NW_007281670.1:215832-222125	0.05	2.77

### Gene-set analysis

**Figure 2:**
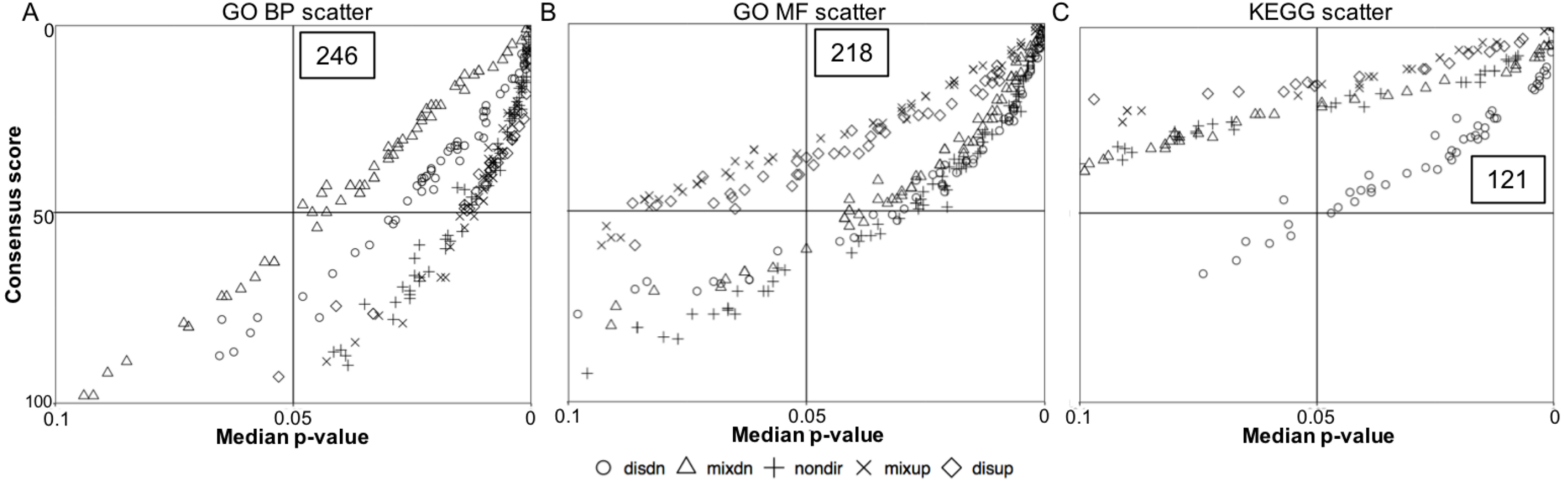
Quadrant scatterplots showing GS analysis results for five directionality classes. The upper right quadrant contains the gene sets with a GS median *p*-value less than 0.05 and a consensus score equal to or less than 50. The total number of the gene sets presented in the upper right quadrant is indicated. (A) GO BP term, (B) GO MF term, and (C) KEGG pathway.

**Figure 3:**
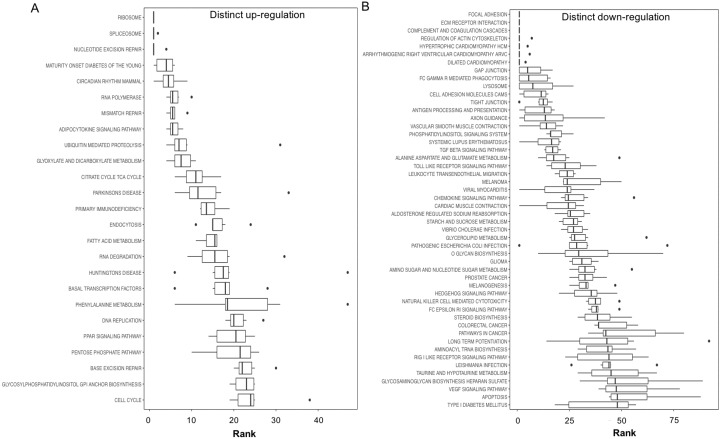
Boxplots showing gene set ranking. Gene sets are ranked based on the GS consensus score and sorted in ascending order (*y*-axis, top down). The box representing the individual ranks from GS analyses with the median value (i.e., GS consensus score) displayed as a vertical line. The dots are outliers. Data are for anoxia induced (A) up-regulated distinct directional and (B) down-regulated distinct directional KEGG pathways.

To determine the anoxia-responsive regulation of distinct pathways identified from transcriptome data of control and 20 hr anoxic sample groups, we employed a GS analysis. This analysis was used to provide an unbiased insight into common pathways under anoxia-responsive transcriptional control that may aid in low oxygen adaptation in the turtle. Scatterplots were used to display the overall GS results for all directionality classes (non-directional, distinct directional and mixed directional) for both GO terms and KEGG pathways (Fig. 2). The upper right quadrant includes the pathways with a median *p*-value less than 0.05 and a consensus score equal or less than 50. As shown in Fig. 2A, a total of 246 median *p*-values (within the five directionality classes) were revealed representing the top significantly enriched GO biological process (BP) terms. Specifically, 49 significantly enriched GO BP terms had a consensus score equal or less than 50 for distinct down-regulation, mixed up-regulation and non-directional regulation classes, while 48 and 51 GO BP terms were enriched for distinct up-regulation and mixed down-regulation classes, respectively (Table S2). Regarding the GO molecular function (MF) terms, 218 median *p*-values were presented in the upper right quadrant (Fig. 2B), including 52 for mixed down-regulation and non-directional classes, 36 for distinct up-regulation, 47 for distinct down-regulation, and 31 for mixed up-regulation (Table S3). Moreover, KEGG pathway analysis showed 121 median *p*-values that were less than 0.05 for the top enriched pathways (Fig. 2C); this included 13, 48, 17, 21, and 22 for distinct up-regulation, distinct down-regulation, mixed up-regulation, mixed down-regulation, and non-directional classes, respectively (Table S4). Figure 3 shows boxplots of the top ranked gene sets from both the up and down-directional classes for KEGG pathway enrichment. Boxplots for the rest of the directionality classes can be viewed in the supplemental material (Figs. S1–S8).

**Figure 4:**
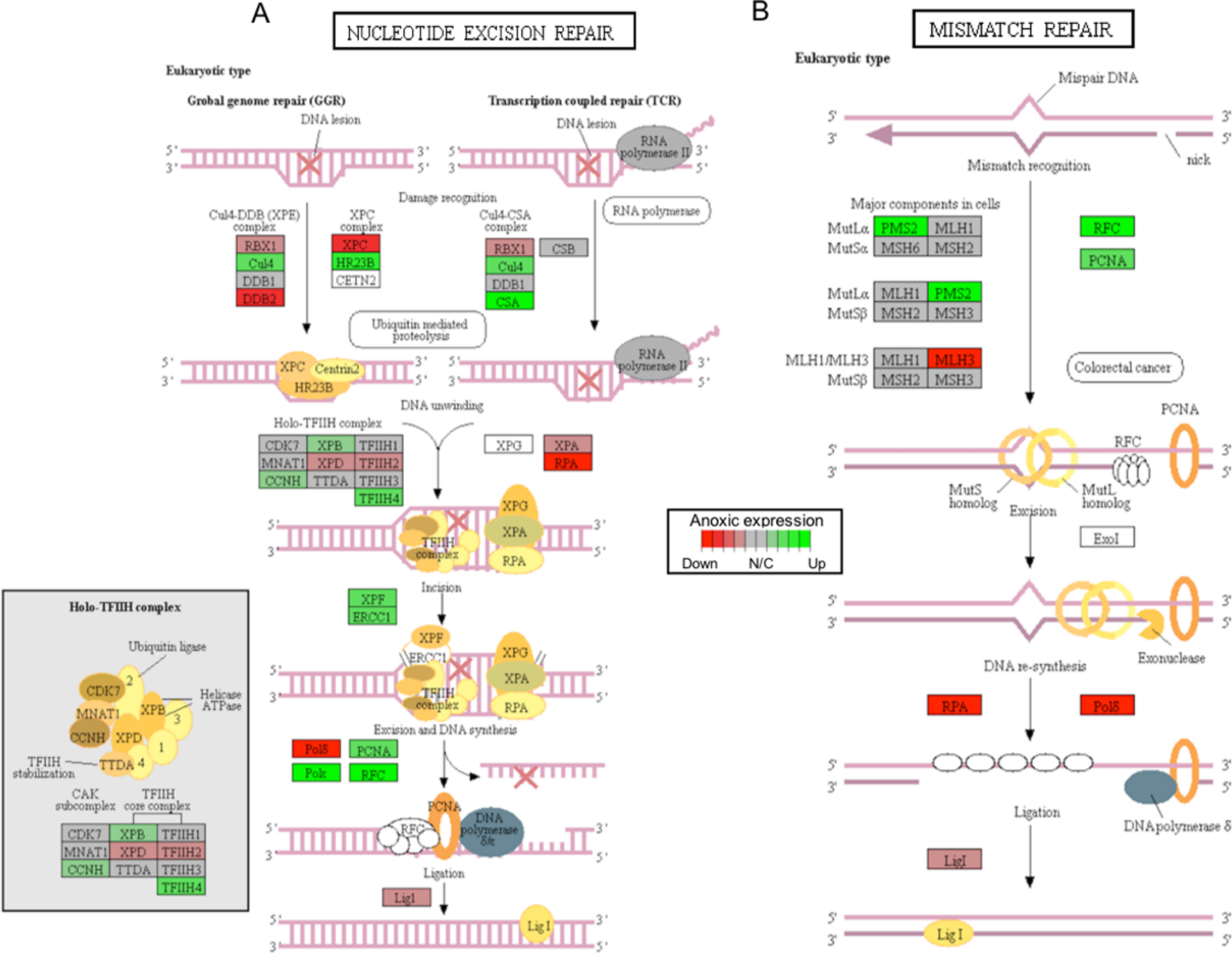
A visual presentation of anoxia-regulated DNA damage signaling and repair KEGG pathways. Pathways are shown for (A) nucleotide excision repair and (B) mismatch repair found to be up-regulated from distinct directional GS analysis. The fold change (logarithmic transformed) for the relevant genes are also plotted with green and red representing increase and decrease, respectively.

**Figure 5:**
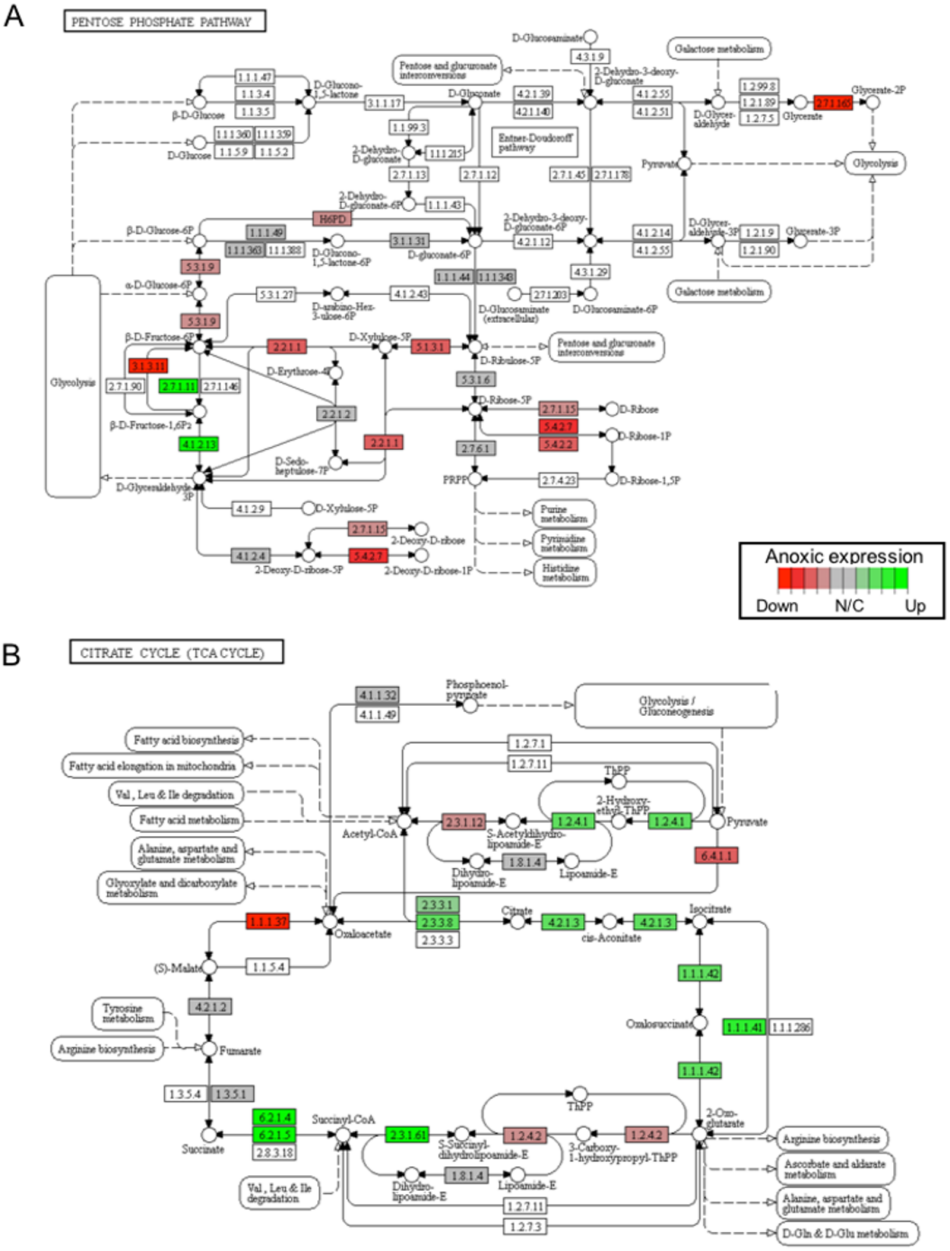
A visual presentation of anoxia-regulated DNA damage signaling and repair KEGG pathways. Pathways are shown for (A) pentose phosphate pathway and (B) citrate cycle KEGG pathways found to be up-regulated from distinct directional GS analysis. The fold change (logarithmic transformed) for the relevant genes are also plotted with green and red representing increase and decrease, respectively.

In this study, 25 KEGG pathways were associated with ≥1.5-fold DE genes found to significantly increase in response to 20 hr anoxia (Fig. 3A). A number of these pathways were related to (1) metabolism, (2) DNA damage and repair, as well as (3) PPAR signaling. The most interesting finding from our RNAseq analysis was that enzyme-encoding genes involved in glucose catabolism, including ‘citrate cycle TCA cycle’ and ‘pentose phosphate pathway’, were found to be enriched in our distinct up-regulated dataset at the transcript level. Details of this and other selected pathways are discussed below in the context of the expected physiological changes and metabolic demands placed upon the liver of anoxic turtles. Opposed to KEGG pathways that were found to significantly increase in response to anoxia, 50 KEGG pathways were associated with genes that were ≥1.5-fold DE down-regulated (Fig. 3B). Among these GS analysis enriched KEGG pathways, a number of pathways were related to (1) growth factor and cytokine signaling and (2) adhesion-related processes. Individual gene regulation patterns of select anoxia-responsive upregulated KEGG pathways are visualized in either DNA damage and repair (Fig. 4) or metabolism-related processes (Fig. 5). Both nucleotide excision repair (Fig. 4A) and mismatch repair (Fig. 4B) are displayed as examples of DNA damage and repair processes that are found to be upregulated in response to anoxia. The pentose phosphate pathway (Fig. 5A) and the citric cycle (Fig. 5B) have been shown as key metabolism-related anoxia-responsive pathways.

## Discussion

It is known that the complete deprivation of oxygen can present several significant, and often fatal, consequences to the cell. Besides the aforementioned stress on metabolism, oxygen deprivation is characterized by an altered redox environment, as well as decreased cellular pH resulting from lactic acid production. In the present study, we used RNAseq to explore the transcriptomic response of *T. scripta elegans* liver to periods of anoxia. As previously discussed, *T. scripta elegans* utilizes a number of different molecular approaches to adapt to a low oxygen environment and is able to avoid anoxia-induced cellular damage through the reprioritization and depression of metabolic processes, such that cellular energy demand can be met by anaerobic metabolism (Storey, 2007). While changes in gene expression have been previously observed in a targeted manner (Krivoruchko & Storey, 2015; Storey, 2007), in this study we attempt to discuss the transcriptome-wide changes that occur in response to anoxia in turtle liver tissue to elucidate the molecular pathways and functions that aid in surviving energy limited systems.

Reflective of the metabolically depressed state of the turtle in response to low oxygen, we found that the majority of genes were found to either decrease in expression or be unchanged from normoxia levels. Of the total 3,105 genes that showed statistically significant changes in gene expression between the two animal groups, 64 genes increased greater than 5-fold when compared to control values (Table 2), while 417 genes decreased to less than 20% of control values (Table S1). Although we did not explore the function of the 2,624 genes that did not fit our criteria to define genes as anoxia-response, we further investigated the 481 anoxia-responsive genes to determine whether anoxic-responsive changes in the transcriptome are reflective of known physiological responses.

Our gene set enrichment approach to identifying pathways that are enriched in anoxia-responsive genes (Fig. 3) revealed a number of pathways for future in-depth analysis. Interestingly, among the top 20 up-regulated pathways, 25% are involved in DNA repair and nucleotide metabolism (nucleotide excision repair, RNA polymerase, mismatch repair, RNA degradation and DNA replication), 25% are metabolism-related (maturity onset diabetes, adipocytokine signaling, glyoxylate and dicarboxylate metabolism, citric acid TCA cycle, and fatty acid metabolism), and 15% protein translation/degradation (ribosome, spliceosome and ubiquitin-mediated proteolysis) (Fig. 3A). Other pathways include circadian rhythm and immunodeficiency. Given the ATP-deprived cellular state that is imposed during anoxia, in a mechanism likely in response aimed towards energy conservation, an even greater number of pathways were found to decrease (Fig. 3B). Overall, our analysis indicates that *T. scripta elegans* may increase cellular capacity to (2) respond to and repair DNA damage, and (2) reprioritize anaerobic metabolism utilizing mechanisms as our top biological processes that may be dynamically regulated by the turtle to respond to anoxia and will be explored in greater detail in the sections below.

### Oxidative stress and DNA damage repair

*T. scipta elegans* is able to survive several weeks of oxygen deprivation while overwintering by severely depressing their metabolic rate. However, upon recovery from anoxia, the re-introduction of oxygen brings about an overproduction of reactive oxygen species (ROS). The turtle is able to tolerate these, typically lethal, high levels of oxidative stress by maintaining an uncharacteristically high basal level of antioxidant defence mechanisms (Willmore & Storey, 1997; Krivoruchko & Storey, 2010).

In this study, the gene expression of nitric oxidase 1 (*NOX1*), a ROS generating enzyme, was determined to increase 5.63-fold following 20 hrs anoxia exposure (Table 2). The physiological function of NOX1 is not currently clear, but given its well-established oxidase activity, it likely involves the production of superoxide. Originally, NOX1 was described as an NADPH oxidase that stimulated mitogenesis (Arnold et al., 2001); however, subsequent studies collectively suggested NOX1 to be a driver of hypertension (Matsuno et al., 2005; Dikalova et al., 2005; Gavazzi et al., 2006). Interestingly, anoxia has been previously found to induce a ten-fold reduction in heart rate and blood flow (Overgaard, Gesser & Wang, 2007). This reduction in cardiac output better matches the requirements of a metabolically depressed state, while blood pressure is still largely maintained through an increase in vascular resistance (Overgaard, Gesser & Wang, 2007). It is possible that NOX1 may have a role in the vasoconstriction displayed in anoxic turtles. In addition to the turtle’s high level of antioxidant defense, our GS analysis suggests that enhanced mechanisms of DNA damage repair may also be an important component of anoxia survival (Fig. 4). Indeed, these repair mechanisms may be enhanced to either maintain genome stability throughout the anoxic exposure or to help protect DNA against the oxidative stress of reperfusion injury.

Critically, ROS is known to produce several forms of DNA damage, including modification of all nucleotide bases, deletions, frame-shifts, strand breaks, chromosomal rearrangements, and intra- and interstrand DNA cross-links (Gonzalez-Hunt, Wadhwa & Sanders, 2018). The *ERCC1* (excision repair cross-complementation group 1) and *XPF* (i.e., ERCC excision repair 4, endonuclease catalytic subunit; a.k.a ERCC4) genes were both found to increase significantly in response to anoxia, among other genes that are involved in nucleotide excision repair, as per KEGG analysis (Fig. 4A; Table S1). Interestingly, together the *ERCC1* and *XPF* genes encode the two subunits of the ERCC1–XPF nuclease. This complex plays an important role in repair of DNA damage and in maintaining genomic stability (Wang et al., 2011). The ERCC1–XPF complex is a core component of nucleotide excision repair and also facilitates inter-strand crosslink repair, pathways of double-strand break repair by homologous recombination and end-joining, as well as telomere length regulation (Wang et al., 2011). Interestingly, a previous study has shown that DNA repair-deficient *ercc1* mutant mice displayed numerous features of accelerated aging and reduced lifespan (Vermeij et al., 2016). Furthermore, *ercc1*-deficient mice have been shown to have increased levels of 8-oxoguanine (i.e., the most common form of DNA lesion resulting from ROS) in their DNA (Hsia et al., 2003). Other central genes involved in DNA damage repair include the sliding clamp proliferating cell nuclear antigen (PCNA) that acts as an important platform for recruiting components of the DNA damage response and that was also found to significantly increase in response to anoxia, as suggested by both DE and GS analyses (Fig. 4B; Table S1) (Mailand, Gibbs-Seymour & Bekker-Jensen, 2013). Together, these findings suggest that DNA damage genes are actively up-regulated to repair oxidative-induced DNA damage, and potentially maintain genome integrity throughout oxidative stress. Indeed, the possibility of anoxia-induced increase in DNA damage repair capacity to help combat increased oxidative stress-related DNA damage has been previously proposed (Krivoruchko & Storey, 2010) and has been further supported by our findings.

### Reprioritization of anaerobic metabolism

In aerobic animals, the complete catabolism of all fuel sources relies on oxidative phosphorylation, in which oxygen plays a crucial role as the final electron acceptor. When oxidative phosphorylation is inhibited, anaerobic pathways of energy production become the sole energy producers of the cell (Storey, 2007). However, simply increasing the rate of anaerobic glycolysis has several limitations, including poor ATP yield, toxic end products (lactic acid), and a quick depletion of fuel sources. Therefore, most aerobic life forms are fairly intolerant of oxygen limitation and sustain significant damage and/or death when subjected to anoxia.

In contrast, *T. scripta elegans* has developed several mechanisms to deal with the challenges posed by utilizing anaerobic glycolysis as their primary ATP producing pathway (Storey, 2007). Although increases in glycolytic rate have been reported for almost all organs of *T. scripta elegans* within the first five hrs of oxygen deprivation, there is a very rapid glycolytic inhibition in the liver occurring within the first hour of anoxia exposure. This reflects the need for glycogenolysis to be directed toward glucose export, supplying the fermentative fuel to be used by other organs (Kelly & Storey, 1988). As a result, a significant metabolic reorganization and reprioritization takes place in the liver in response to anoxia. Our RNAseq analysis has identified a number of differentially expressed metabolic enzymes and anoxia-responsive metabolic pathways. For example, significant alterations in the expression of a number of glycolytic enzymes in response to 20 hrs of anoxia exposure were suggested by the RNAseq DE results. This includes a 17-fold increase in the glucokinase binding protein, *GKRP* (Table 2); encoding a protein that modulates glucokinase (GK; 2.7.1.2) activity and location of gluconeogenesis as glucose levels decline. In conditions of low glucose, GKRP relocates GK to the nucleus where it is inactive. Increasing amounts of glucose prompts the GKRP to rapidly release GK to return to the cytoplasm (Choi et al., 2013). Such a mechanism may help to dynamically control liver glucose metabolism in response to anoxia.

The transcript levels of glycolytic enzymes, phosphofructokinase (*PFK*; 2.7.1.11) and aldolase (*ALDO*; 4.1.2.13), were also found to significantly increase in response to 20 hrs anoxia (Fig. 5A). Specifically, *PFK* significantly increased 2.77-fold, whereas *ALDO* increased 4.89-fold when compared to normoxic control values. Although changes in transcript abundance are not necessarily reflective of changes in enzyme function or activity, previous studies have reported significant increases in the activities of these two enzymes in response to anoxia in *T. scripta elegans* liver. One study documented significant changes in the enzyme activity of PFK upon prolonged exposure to anoxia, demonstrated a 3-fold decrease in the I_50_ value (i.e., the inhibitor concentration reducing enzyme velocity by 50%) for citrate, and found a 1.5-fold increase in the K_m_ value (i.e., the substrate concentration producing half maximal enzyme activity) for ATP during anoxia (Brooks & Storey, 1989). Corroborating our RNAseq analysis, the protein expression of ALDO has been previously shown to significantly increase by 1.6-fold in response to 20 hrs anoxia in *T. scripta elegans* liver (Dawson, Biggar & Storey, 2013). In that study, the increase in fructose-1,6-bisphosphate (FBP) levels known to occur during anoxia were found to be accompanied by both an increase in endogenous ALDO protein expression, along with higher affinity for FBP in turtle liver. Another study also reported that the relative levels of FBP in liver doubled after only 1 hr of anoxia in *T. scripta elegans* (Kelly & Storey, 1988). When considering the presence of large liver glycogen stores and general increase in anaerobic glycolysis seen during anoxic exposure, the increase in FBP levels suggests the regulatory importance of aldolase in anoxic liver tissue.

Our RNAseq analysis also identified a significant increase in the gene expression of citrate cycle enzymes that support succinate production, which was enriched by the GS KEGG analysis (Fig. 5B). In this regard, it has been established that anaerobic glycolysis with lactate as the end product has two limitations: (1) a low yield of ATP per glucose catabolized, and (2) significant cellular acidification. Although the turtle has unique mechanisms to help deal with the latter, several alternative end products to lactate have appeared in anoxia-tolerant species that provide enhanced ATP yield per glucose catabolized. Collectively, these include ethanol, alanine, succinate, propionate, and acetate (Muller et al., 2012). For marine invertebrates, during prolonged anoxia, carbon from glycolysis is directed into the reactions of succinate synthesis. The energy yield of direct fermentation of glucose to succinate yields a molecule of GTP per molecule glucose in addition to the two molecules that result from lactate production. Although a mechanistic exploration into the enzymatic production of succinate as an anoxia end product of glucose catabolism in *T. scripta elegans* has not yet been completed, previous studies have explored alternative glycolytic end products in response to prolonged anoxia in *C. picta bellii* (Buck, 2000). In response to a 28-day anoxic dive at 5 °C, succinate, alanine, and lactate concentrations were all found to increase significantly in liver tissue. Relative to levels of lactate, this study found that levels of succinate and alanine increased by 2 and 0.9% in liver, 0.3 and 0.04% in blood, and 0.6 and 0.07% in heart, respectively. Together, these findings suggest a role for succinate production in turtle liver tissue as a possible glycolytic alternative to lactate. Indeed, our RNAseq analysis supports the hypothesis that succinate accumulation may be a component of anaerobic metabolism (Fig. 5B). For example, at the level of the transcriptome, our analysis found a significant increase in the anoxic expression of succinyl-CoA-synthetase (SCS), which was found to be elevated 1.62-fold in comparison to normoxia values. Critically, the SCS enzyme is responsive for the catalysis of the reversible reaction of succinyl-CoA to succinate and the associated production of GTP. Collectively, these findings support the possibility of glucose catabolism to succinate as a possible alternative fate to lactate, yielding greater energy yield but is likely a minor anaerobic end-product compared to lactate.

## Conclusion

Although much is known about the physiological responses of anoxic exposure in the turtle, *T. scripta elegans*, studies are beginning to elucidate the molecular underpinnings that coordinate these adaptations. As research begins to utilize strategies of transcriptomic and proteomic-wide analysis to explore models of metabolic depression and extreme environmental adaptations, we will begin to better understand the molecular mechanisms that are in place to support survival. For example, this study highlighted a number of anoxia-responsive molecular pathways that are likely critical to navigating anoxia survival, such as DNA damage repair and metabolic reprogramming. Furthermore, the possibility of succinate production being utilized as an additional energy production pathway in addition to lactate metabolism was also highlighted as part of this study. Ultimately, this study has set the stage for future research to navigate and document the many insights that have been uncovered.

##  Supplemental Information

10.7717/peerj.8144/supp-1 Supplemental Information Enrichment boxplots

10.7717/peerj.8144/supp-2 Table S1 Raw RNAseq statistics

10.7717/peerj.8144/supp-3 Table S2 GO biological process enrichment statistics

10.7717/peerj.8144/supp-4 Table S3 GO molecular function enrichment statistics

10.7717/peerj.8144/supp-5 Table S4 KEGG pathway enrichment statistics

## Abbreviations

ATP: adenosine nucleotide tri-phosphate
BP: biological process
DE: differential expression
FDR: false discovery rate
FPKM: fragments per kilobase of transcript per million mapped reads
GFF: general feature format
GO: gene ontology
GTP: guanine nucleotide tri-phosphate
GS: gene set
GSEA: gene set enrichment analysis
KEGG: Kyoto encyclopedia of genes and genomes
MF: molecular function
RNAseq: RNA sequencing
TCA: tricarboxylic acid cycle
PAGE: parametric analysis of gene set enrichment
ROS: reactive oxygen species
ERCC1: excision repair cross-complementing group 1
SCS: succinyl coA synthetase
GK: glucokinase
GKRP: glucokinase regulatory protein
FBP: fructose-1,6-bisphosphate

## Additional Information and Declarations

### Data Availability

The following information was supplied regarding data availability: Sequence reads are available at NCBI SRA: SRP187832.
