# Supplementary material for: Navigating oxygen deprivation: liver transcriptomic responses of the red eared slider turtle to environmental anoxia
Source: PeerJ. 2019 Nov 26;7:e8144. doi: 10.7717/peerj.8144 (PMC6883951; doi:10.7717/peerj.8144)
Supplement: Supplemental Information [file peerj-07-8144-s001.pdf]

Supplemental figures

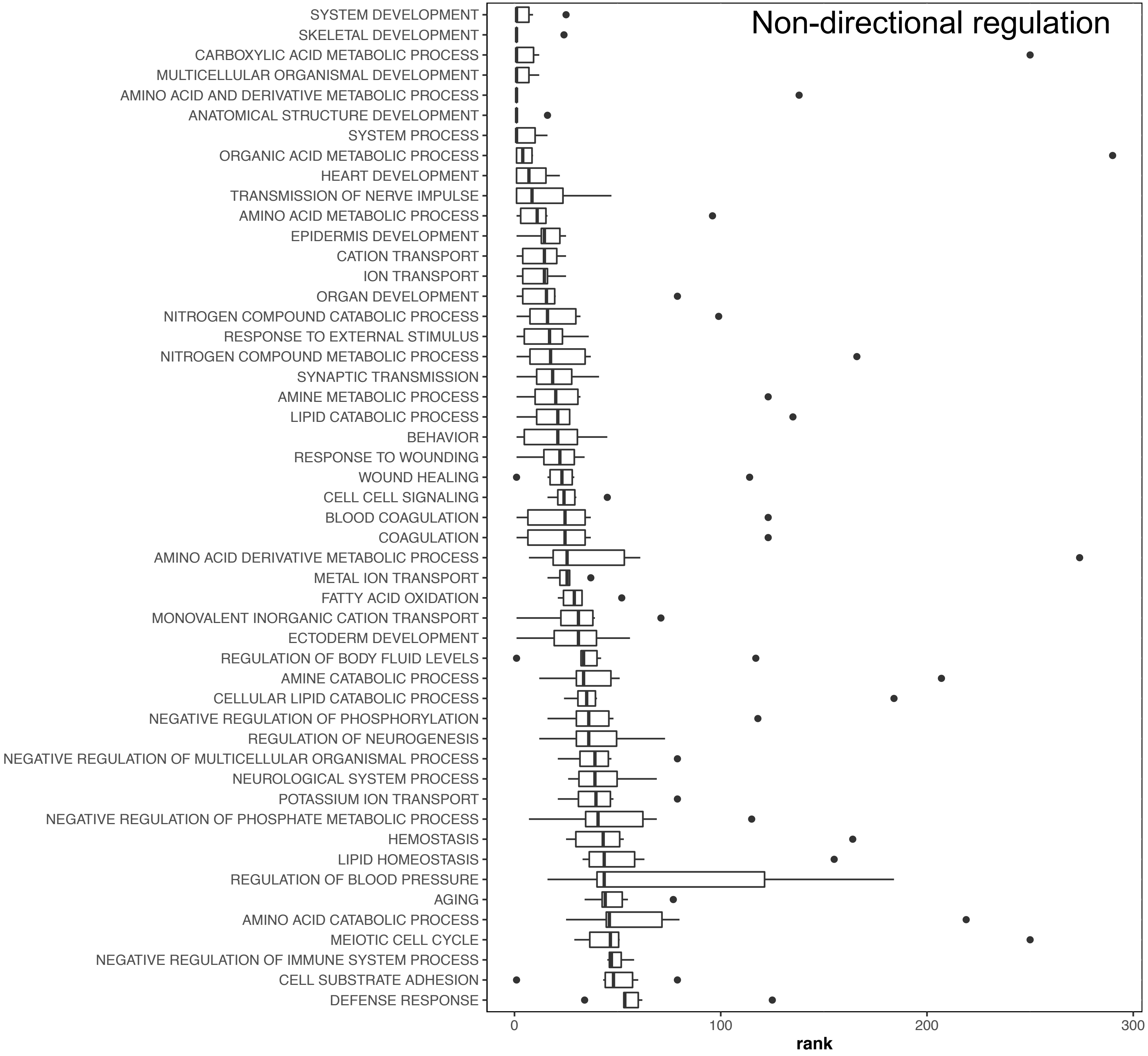

Figs S1. Gene Ontology Biological Process boxplot – non-directional regulation.

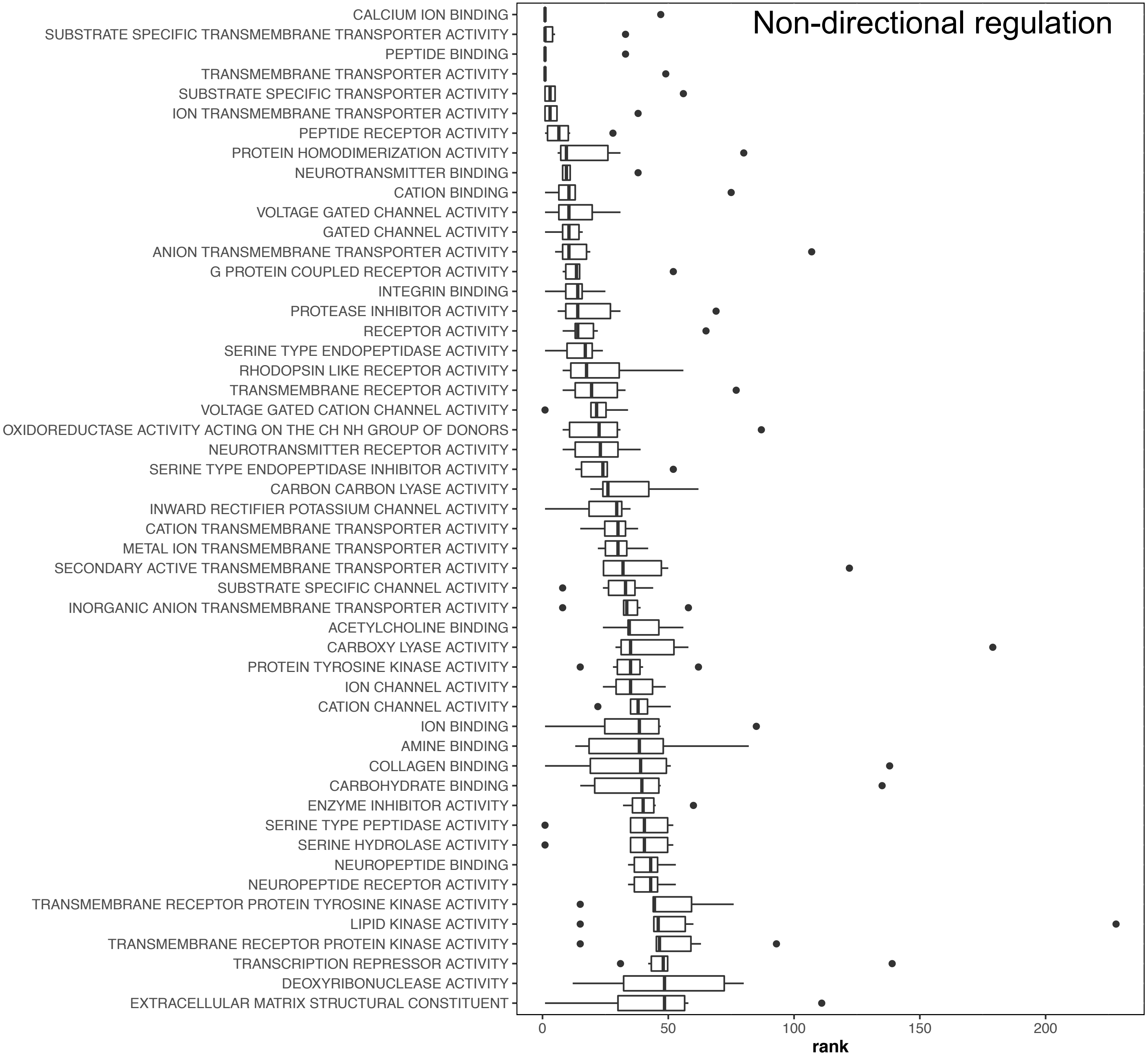

Figs S2. Gene Ontology Molecular Function boxplot – non-directional regulation.

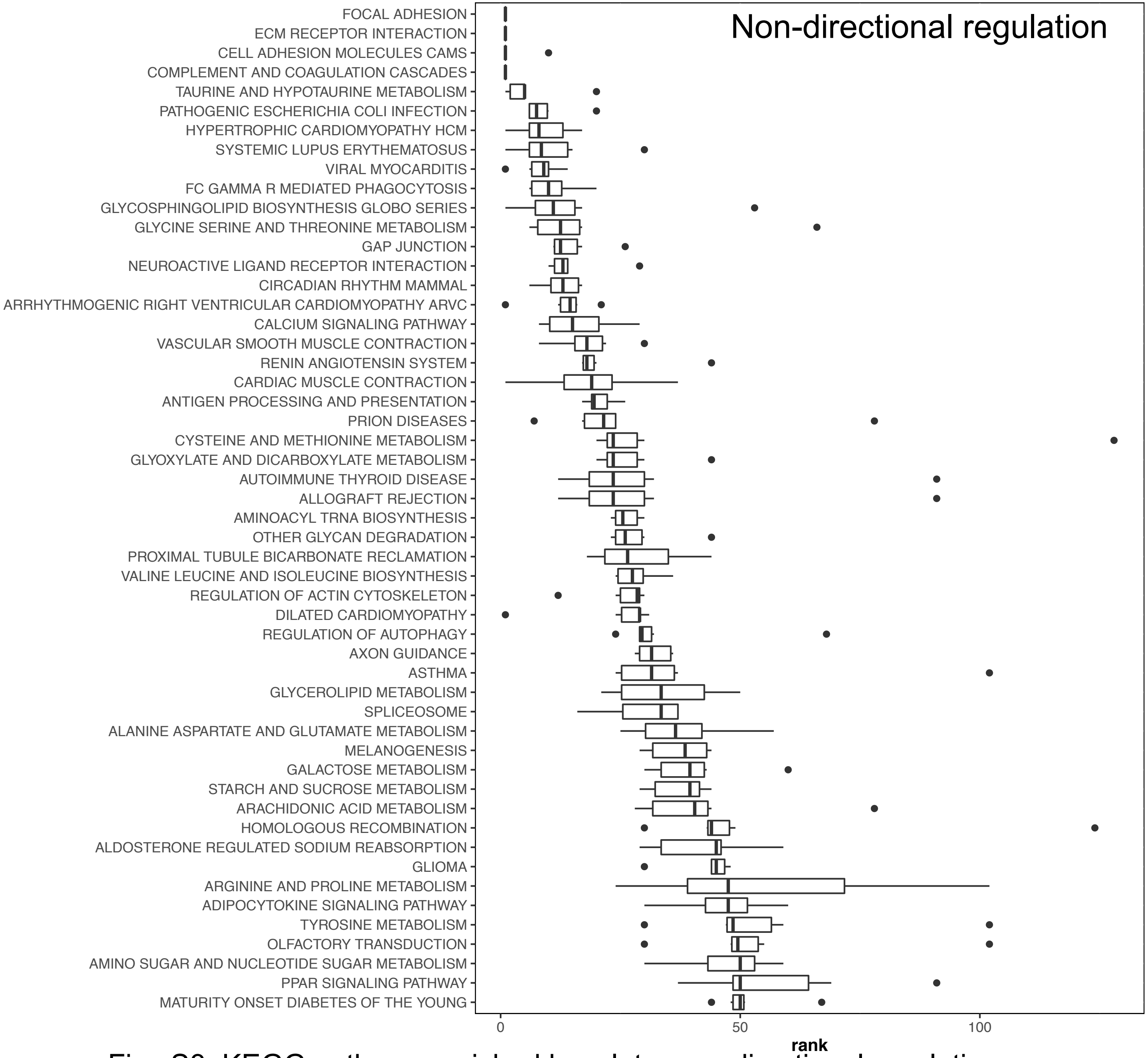

Figs S3. KEGG pathway enriched boxplot – non-directional regulation.

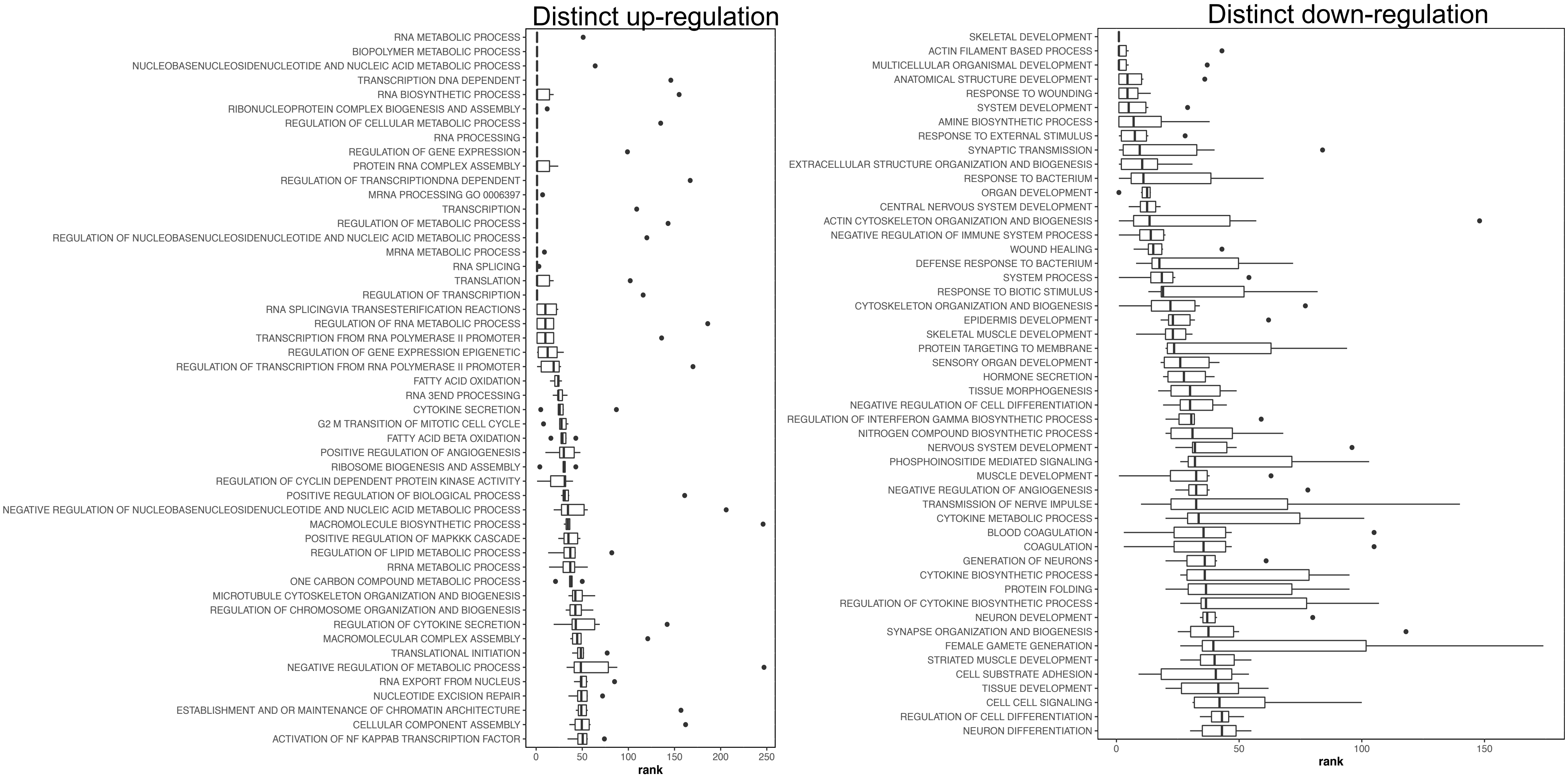

Figs S4. Gene Ontology Biological Process boxplot – distinct-directional regulation. (A) distinct up-regulated processes. (B) distinct down-regulated processes.

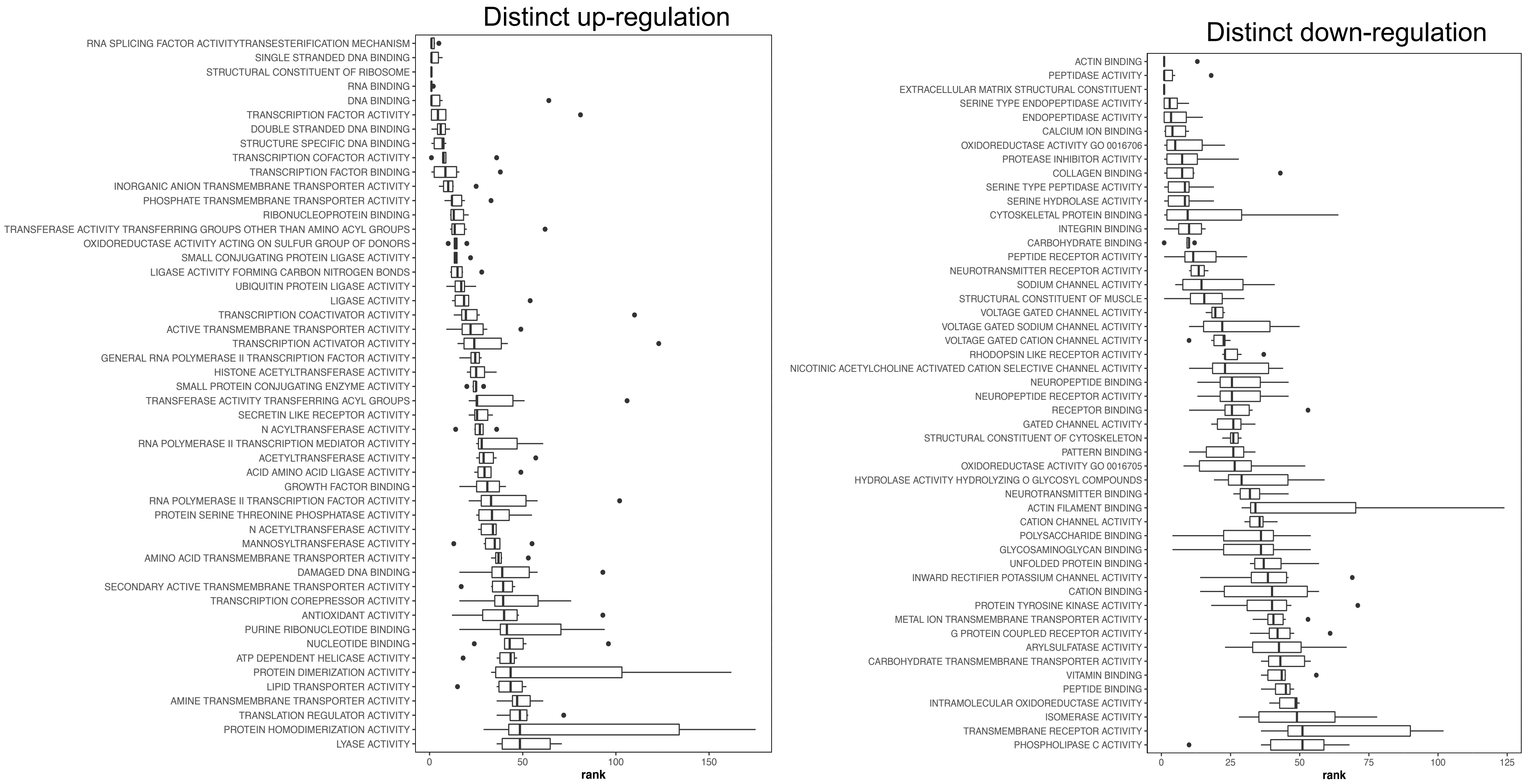

Figs S5. Gene Ontology Molecular Function boxplot – distinct-directional regulation. (A) distinct up-regulated processes. (B) distinct down-regulated processes.

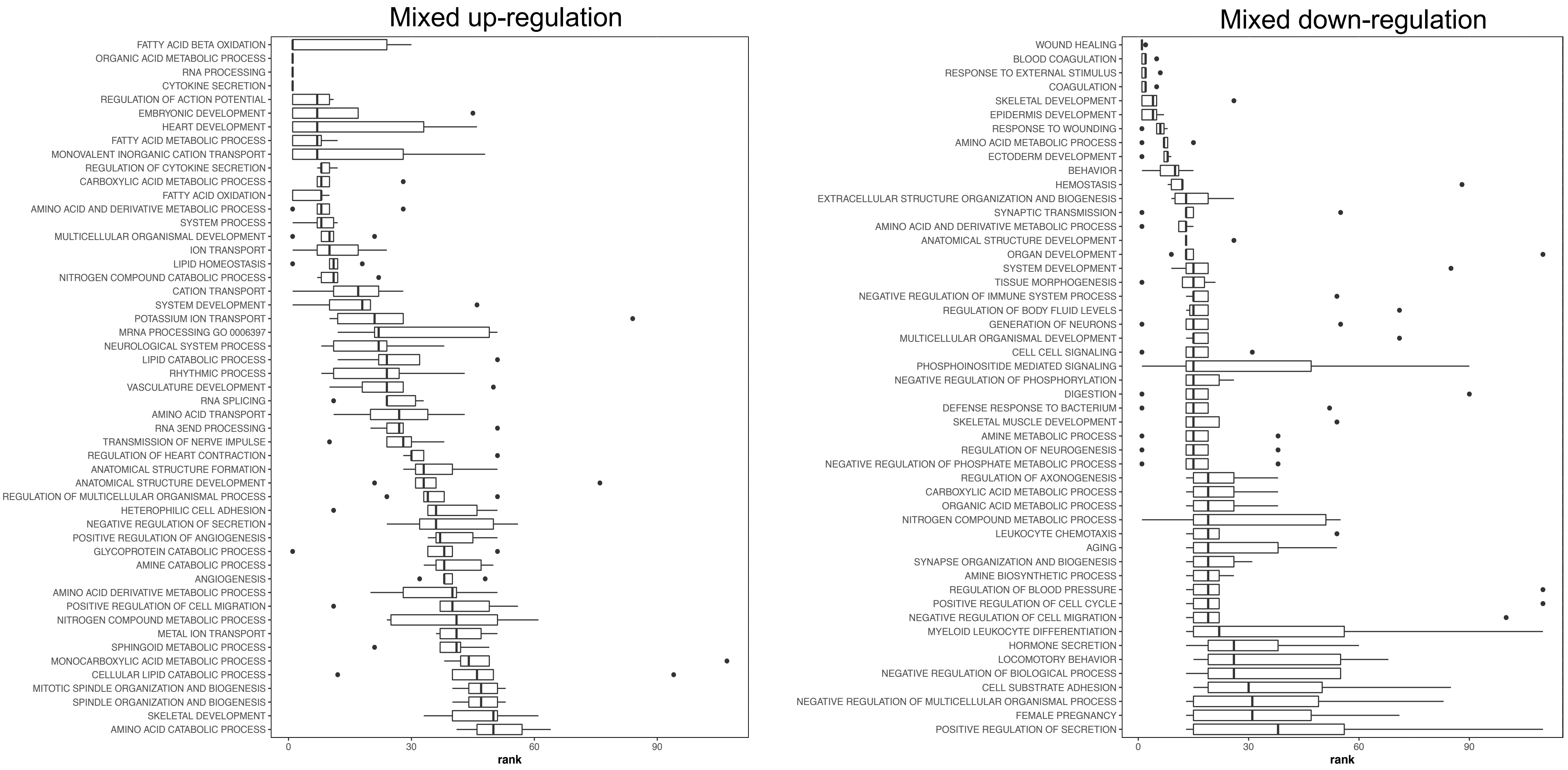

Figs S6. Gene Ontology Biological Process boxplot – mixed-directional regulation. (A) mixed up-regulated processes. (B) mixed down-regulated processes.

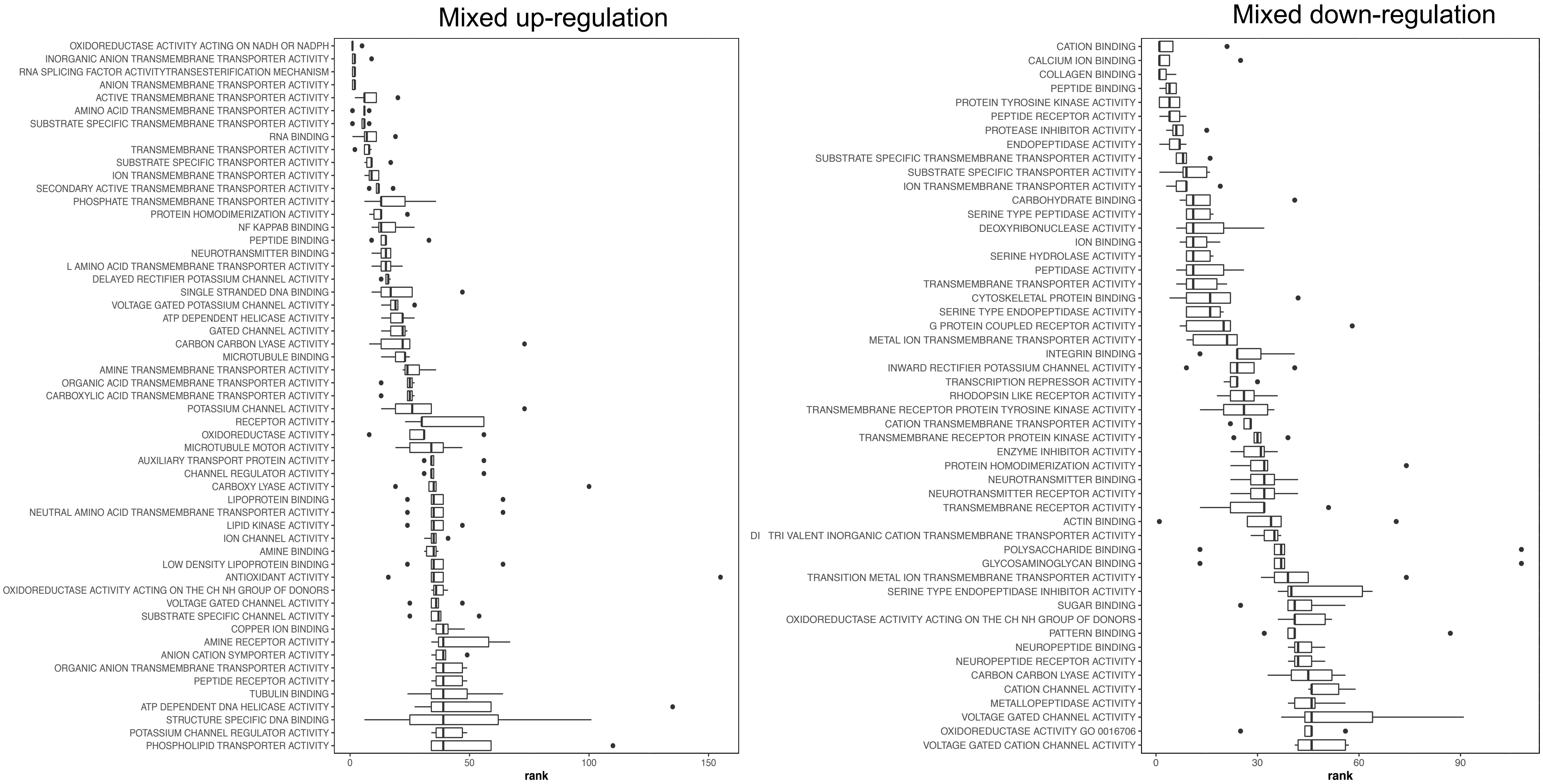

Figs S7. Gene Ontology Molecular Function boxplot – mixed-directional regulation. (A) mixed up-regulated processes. (B) mixed down-regulated processes.

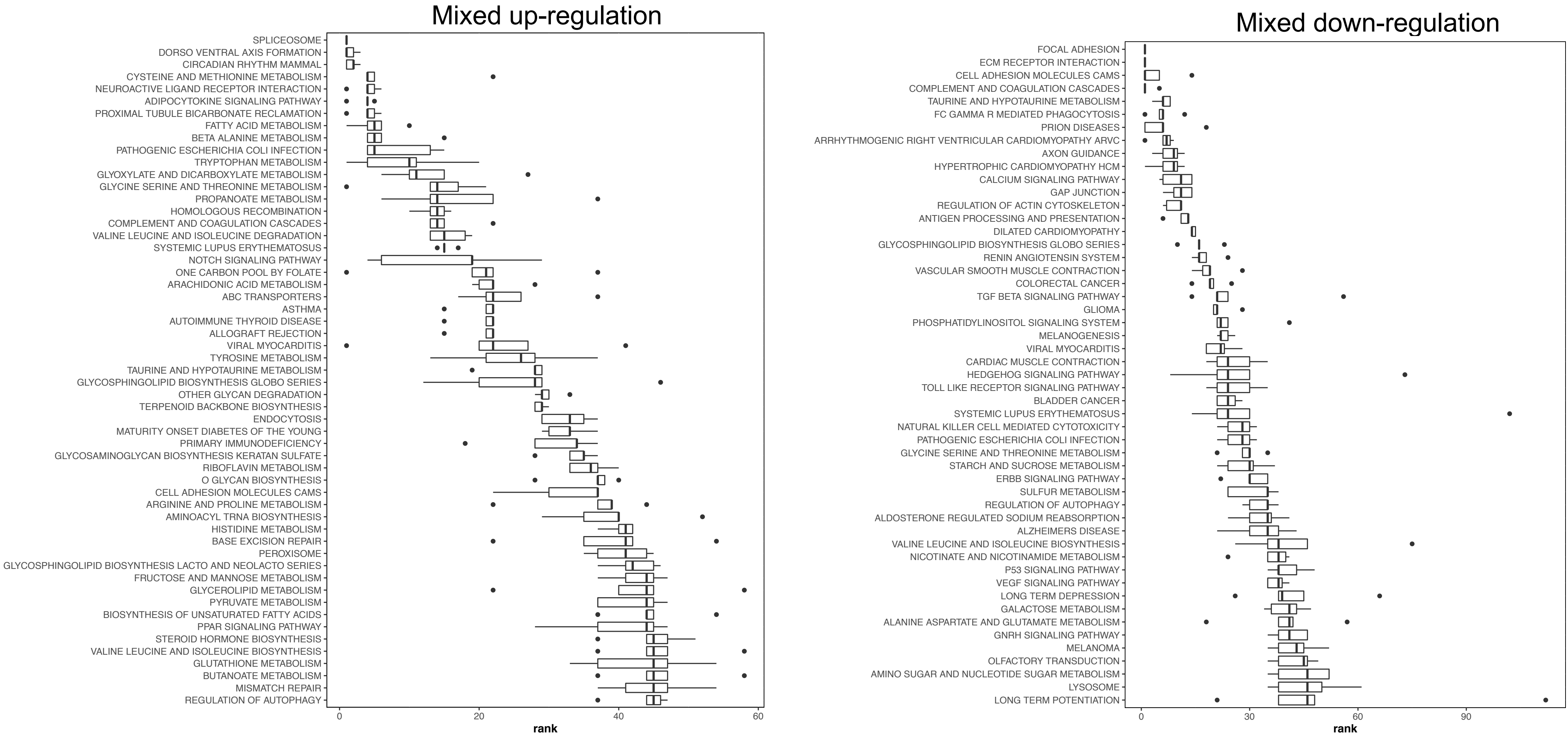

Figs S8. KEGG pathway enriched boxplot – mixed-directional regulation. (A) mixed up-regulated KEGG pathways. (B) mixed down-regulated KEGG pathways.
